# Thoracic Spinal Cord Compression Secondary to Metastatic Papillary Thyroid Carcinoma: An Unusual Oncological Phenomenon

**DOI:** 10.7759/cureus.24206

**Published:** 2022-04-17

**Authors:** Eltaib A Saad, Monzer Abdalla, Abdalaziz M Awadelkarim, Osama Elkhider, Mohamed Agab, Akram Babkir, Isra Idris, Dorota Filipiuk

**Affiliations:** 1 Internal Medicine, AMITA Health Saint Francis Hospital, Evanston, USA; 2 Internal Medicine, Wayne State University Detroit Medical Center, Detroit, USA; 3 Pediatrics, Woodhull Medical Center, New York, USA; 4 Pathology, AMITA Health Saint Francis Hospital, Evanston, USA

**Keywords:** metastatic papillary thyroid cancer, cord compression, spinal metastasis, hematogenous spread, papillary thyroid carcinoma

## Abstract

Hematogenous spread is fairly an unusual feature for papillary thyroid carcinoma (PTC) in comparison to follicular thyroid carcinoma (FTC). Thoracic spinal metastasis with complicating cord compression is an even rarer manifestation of PTC that was reported in a limited number of cases in the literature. Herein we present a 65-year-old female with a history of PTC on current radiotherapy, status post attempted surgery due to significant tumor burden and intraoperative bleeding, presented with a one-week history of rapidly progressive bilateral lower extremities weakness. Physical examination revealed paraplegia of both lower extremities with areflexia and a sensory level equivalent to the upper thoracic vertebrae. Urgent imaging depicted destructive epidural lesions at T1-T3 vertebrae with thoracic cord compression. Emergent laminectomy and debulking of these lesions were undertaken. Histopathological examination confirmed metastatic PTC. The patient proceeded to further treatment with radiotherapy following her successful neurological recovery. Thoracic vertebral metastasis is an unusual oncological phenomenon of PTC. Metastatic PTC should be considered in patients with a current or remote history of PTC who present with thoracic cord compression. Our case demonstrates that multidisciplinary management is the key to achieving a better outcome for metastatic PTC with thoracic cord compression.

## Introduction

Papillary thyroid carcinoma (PTC) is the most common type of differentiated thyroid carcinoma (DTC) (75%), followed by follicular thyroid carcinoma (FTC) [1]. Generally, PTC spreads locally via the lymphatic system, and the hematogenous dissemination is quite unusual compared to FTC. The latter tends to metastasize through the bloodstream to distant organs, including the lungs, brain, and bones [2, 3]. Spinal vertebrae constitute the main site of bony metastasis of TC [3, 4]. The reviewed literature reported that 3% of patients with DTC have spinal metastasis [4]. FTC is more likely to metastasize to the spine than PTC because of the tendency of hematogenous spread of FTC (7% to 28% and 1.4% to 7%, for FTC and PTC, respectively) [4]. Herein, the authors present an unusual case of thoracic cord compression secondary to metastatic PTC in an elderly female patient with a history of synchronous PTC that was under radiotherapy treatment.

## Case presentation

A 65-year-old African American female with a history of papillary thyroid carcinoma (PTC) under current radiotherapy treatment presented to our emergency department (ED) with a one-week history of progressive bilateral lower extremities weakness and loss of sensation associated with upper back pain. Her condition progressed rapidly to the extent that she had to hold on to furniture to ambulate, and she had multiple weakness-related falls at home. She denied bowel or bladder incontinence.

A review of the systems was negative for fever or rigors, cough, shortness of breath, bowel habit changes, hematochezia or melena, and urinary symptoms. Past medical history was relevant for essential hypertension for which she was regularly taking amlodipine. She had been diagnosed with well-differentiated PTC six weeks prior to her index presentation to our ED. Histopathology report demonstrated a follicular variant PTC with capsular and vascular invasion along with typical nuclear changes of PTC. Immunohistochemistry staining was positive for TTF1 and thyroglobulin. The preoperative staging was cT4aN1M0. Total thyroidectomy with neck dissection was unsuccessfully attempted at a tertiary oncology center due to extensive tumor burden and significant intraoperative bleeding. The postoperative imaging (around five weeks before presenting to the ED presentation) with a contrast-enhanced computed tomography (CT) of the neck revealed interval worsening progression of the size of the neoplastic thyroid mass with invasion of the anterior chest wall eroding the upper sternum. Radiation oncology consultation at that time recommended neoadjuvant radiotherapy for downstaging of the tumor burden, and the patient underwent the first two sessions of external beam radiation with fairly good tolerance in the last week prior to her index presentation to our facility.

**Figure 1:**
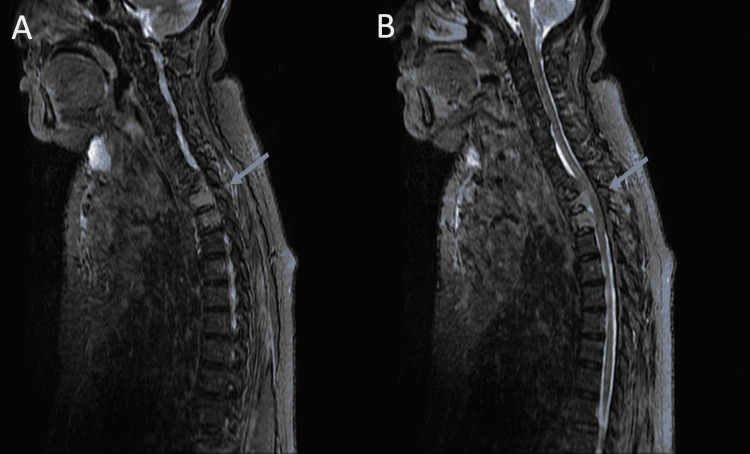
(A&B) Sagittal images of T2-weighted MRI of the thoracic spine with contrast. Figure A with radial blue arrow showing infiltrating and destructive epidural lesions at T2 and T3 vertebral bodies in keeping with metastatic disease. Figure B with radial blue arrow pointing towards thoracic cord segment compression at T2 and T3 levels caused by likely metastatic lesions.

Initial evaluation at the emergency department revealed a vitally stable but anxious elderly woman. The neck examination revealed an enlarged thyroid gland with an unpalpable lower border. The neurological exam demonstrated bilateral paraplegia with areflexia and a loss of light touch and pain sensation up the level of the upper thoracic vertebrae. The anal examination revealed intact anal tone without perineal numbness. Motor and sensory examination of both upper extremities were essentially normal. Upper thoracic cord compression was suspected. Urgent magnetic resonance imaging (MRI) of the thoracic spine depicted large, infiltrating, and destructive masses within the T1, T2, and T3 vertebral bodies with significant cord compression at the T2 and T3 levels (Figure 1).

The patient was commenced on high-dose intravenous steroids (10 mg of dexamethasone stat dose and then every 6 hours) per neurosurgical consultation. She underwent an emergent decompressive laminectomy with debulking resection of the destructive epidural lesions.

**Figure 2:**
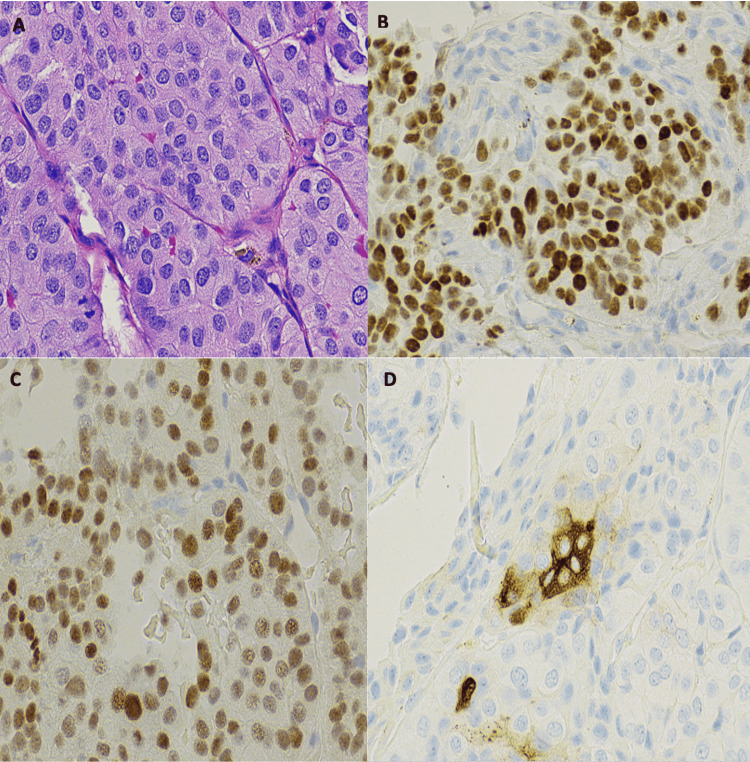
(A) Hematoxylin & Eosin (H&E x40) revealed metastatic papillary thyroid carcinoma of a follicular variant. (B-D) demonstrates positive immunohistochemistry staining with TTF, PAX8, and Thyroglobulin stains, respectively.

Pathological examination of the surgical biopsy demonstrated positive staining for PAX-8, TTF-1, and thyroglobulin with intranuclear groove, nuclear membrane irregularity and central chromatin, all features were in keeping with the diagnosis of metastatic papillary thyroid carcinoma of follicular variant that was typically consistent with the original thyroid tumor’s biopsies performed on the initial diagnosis (Figure 2).

The postoperative course was largely uneventful, and the patient made a remarkable neurological recovery with physiotherapy. She was scheduled for completion radiotherapy two weeks postoperatively. The patient was discharged into a rehabilitation facility for ongoing physiotherapy. A whole-body I-131 scan was performed after the 4th session of postoperative radiotherapy and showed uptake in the thyroid bed with retrosternal tissues extension without evidence of osseous metastasis.

## Discussion

The reviewed literature reported that distant metastases from well-differentiated thyroid carcinoma (TC) occur in up to one-fifth (20%) of total TC cases, and it is considered the most common cause of thyroid cancer-related mortality [5, 6]. A 10-year survival in one series of TC patients with osseous and non-osseous metastasis ranged between 13-42% [7]. About 3% of patients with TC have osseous spinal metastasis [4], however, compared with all other secondary spinal metastasis, metastatic TC has the most favorable prognosis [5]. Vertebral involvement can occur due to direct spreading of TC with subsequent thyroid tissues’ extension into the spinal canal. Indirect vertebral involvement can also occur through the hematogenous spread through the distribution of the segmental medullary branches to the axial skeleton, with FTC much more frequently than PTC (7% to 28% and 1.4% to 7%, respectively) [2].

Most of the spinal metastases from TC affect the thoracic vertebrae (as shown in our case) followed by the lumbar and cervical vertebrae through valveless Bateson’s venous plexus. Cord compression secondary to metastatic deposits is more common in the upper thoracic segments due to the smallest ratio of the spinal canal to the spinal cord at this vertebral level [8].

The clinical features of spinal metastasis from TC include cord compression (45%), radiculopathy (12%), and back pain as a chief presenting complaint (28%). Less than one-fifth of spinal metastasis was asymptomatic that has been detected during routine investigations at initial diagnosis or subsequent surveillance. Furthermore, about one-third (35%) of spinal metastasis manifested as an initial presentation of TC [3]. Interestingly, spinal metastasis with a thoracic cord compression was a culprit of the recurrence of TC following ten years of hemithyroidectomy of a PTC, and hence metastatic TC should be considered as a differential diagnosis for secondary bone cancers, even after years following original TC surgery [9].

In a cohort of patients with spinal metastasis of TC, it was found that spinal metastasis (SM) was the first manifestation of FTC as a synchronous tumor, compared to PTC where SM presented after initial TC diagnosis in a metachronous pattern [3], as demonstrated in our patient. Histopathological sub-analysis of metastatic PTC revealed a follicular pattern, this variant was associated with capsular invasion, angioinvasion, and hematogenous spread with distant metastases than other PTC variants [8].

Management of spinal metastasis of TC should involve a multidisciplinary team comprising spinal surgeons, clinical oncologists, and radiation-oncology specialists to achieve a better outcome [5]. Therapeutic interventions for metastatic disease are aimed to restore the spinal cord integrity, relieve local pain, and reverse neurological compromise [3, 5]. There are a variety of options including a combination of decompressive surgery with debulking for cord compression or intractable pain, minimally invasive vertebroplasty for osteolytic bone lesions without spinal instability, radiotherapy with external beam radiation, radioiodine ablation therapy, and medical therapy with bisphosphonates [2, 3]. It is interesting to note that metastatic TC lesions in the spine are usually hyper-vascular with rich capillary beds, therefore a preoperative selective arterial embolization of these lesions can minimize intraoperative blood loss as demonstrated in one case [10].

Favorable prognostic factors of metastatic spinal TC from the previous series were younger patient age, well-differentiated papillary or follicular subtypes of the tumor that were responsive to radioiodine ablation therapy, and radioiodine avidity of the primary and metastatic neoplasms [4].

## Conclusions

Thoracic vertebral metastasis with cord compression is an unusual oncological phenomenon of PTC. Metastatic PTC should be considered in patients with a current or remote history of PTC who present with cord compression. Our case demonstrates that multidisciplinary management is the key to achieving a better outcome for metastatic PTC with thoracic cord compression.
